# Behavioral cross-sensitization between testosterone and fenproporex in adolescent and adult rats

**DOI:** 10.1590/1414-431X20176388

**Published:** 2017-11-17

**Authors:** C.Q. Conceição, S.A. Engi, F.C. Cruz, C.S. Planeta

**Affiliations:** 1 Laboratório de Neuropsicofarmacologia, Faculdade de Ciências Farmacêuticas, Universidade Estadual Paulista, Araraquara, SP, Brasil; 2 Programa Interinstitucional de Pós-Graduação em Ciências Fisiológicas, Universidade Federal de São Carlos/Universidade Estadual Paulista, PIPGCF UFSCar/UNESP, São Carlos, SP, Brasil; Universidade Estadual Paulista, Universidade Estadual Paulista, Brasil

**Keywords:** Behavioral sensitization, Fenproporex, Testosterone, Rats

## Abstract

The abuse of psychoactive drugs is considered a global health problem. During the last years, a relevant number of studies have investigated the relationship between anabolic-androgenic steroids (AAS) and other psychoactive drugs. AAS, such as testosterone, can cause a dependence syndrome that shares many features with the classical dependence to psychoactive substances. Pre-clinical evidence shows that there are interactions between testosterone and psychoactive drugs, such as cocaine. However, few studies have been performed to investigate the effect of repeated testosterone treatment on behavioral effects of amphetamine derivatives, such as fenproporex. The purpose of the present study was to investigate the effects of repeated testosterone administration on fenproporex-induced locomotor activity in adolescent and adult rats. Adolescent male Wistar rats were injected with testosterone (10 mg/kg *sc* for 10 days). After 3 days, animals received an acute injection of fenproporex (3.0 mg/kg *ip*) and the locomotor activity was recorded during 40 min. Thirty days later, the same animals received the same treatment with testosterone followed by a fenproporex challenge injection as described above. Our results demonstrated that repeated testosterone induced behavioral sensitization to fenproporex in adolescent but not in adult rats. These findings suggest that repeated AAS treatment might increase the dependence vulnerability to amphetamine and its derivatives in adolescent rats.

## Introduction

Psychoactive drug abuse is a global health problem. The United Nations Office on Drugs and Crime (1) reported that 149 to 272 million people between 15 and 64 years used some illicit drug at least once in the year before the report.

Amphetamine-type stimulants (ATS) are ranked as the world's most popular illicit drug, with about 33 million users (1). ATS are psychostimulant molecules that are used in the treatment for attention deficit disorder, narcolepsy and obesity (2). Between 2006 and 2009, Belgium and Brazil were the principal fenproporex manufacturer countries. In 2009, Brazil was the principal importer of this anorectic drug and in 2010 only Brazil manufactured fenproporex (3).

In Brazil, there are no official reports about the illicit use of anabolic androgenic steroids (AAS), but users are mainly men between 18 and 34 years old (4). In the last years, AAS have been used for enhancing athletic performance and/or improving body image (5). Importantly, there is evidence that the dosage used for this purpose is 10 to 100 times higher than medical prescriptions (5).

ATS are derived from phenethylamines and are similar to adrenaline. Thus, β-phenethylaminics, such as fenproporex, increase the noradrenergic and dopaminergic neurotransmission in the central nervous system, increasing the intracellular dopamine concentration in the limbic system (6–8). The principal central mechanism of fenproporex is similar to amphetamine, increasing the dopamine levels through three main mechanisms: blocking the dopamine transporter (inhibiting the dopamine reuptake), increasing dopamine release, and acting as a monoamine oxidase inhibitor (9). It is well known that dopamine causes neuroadaptations in the limbic system that are related to addiction behaviors (10).

AAS are a hormone family that includes testosterone and its analogues (11). In most cell targets, testosterone acts through its active metabolite, dihydrotestosterone. Testosterone and dihydrotestosterone can modify gene transcription through interaction with membrane and intracellular receptors modulating many physiological systems (12), such as the limbic system (13).

Recent studies have shown that AAS are associated to many psychiatric disorders (5,14). Repeated use of AAS cause addiction and withdrawal syndrome as occurs with the repeated use of psychoactive drugs, such as cocaine and amphetamine (15,16). Evidence supports that AAS act as a positive reinforcement in animal self-administration models (5,11,17,18). Animal studies suggest that AAS act in the reward system similarly to classic drug abuse (19).

Recently, an increasing number of studies are investigating the relationship between AAS and other psychoactive substances. There is a significant body of evidence showing the concomitant use of AAS and tobacco, alcohol, and illicit drugs (17,20,21). Studies demonstrate that AAS users also self-administer stimulant and anorectic drugs, such as amphetamines and their derivatives, to sustain a fine self-image (5).

Despite the importance, little is known about the effects of chronic testosterone administration on psychoactive substance behavior, including ATS. In fact, previous results from our laboratory showed that the chronic treatment with testosterone caused an increase in the cocaine stimulant effect in adolescent and adult rats, and locomotor cross-sensitization in adolescent rats (22). Therefore, a question to be answered is whether chronic testosterone use could promote cross-sensitization to amphetamine derivatives in adolescent and adult rats.

## Material and Methods

### Subjects

Male Wistar rats on postnatal day (PND) 21, obtained from the animal breeding facility of the Universidade Estadual Paulista (UNESP) were used. Groups of 4-5 animals were housed in plastic cages [32 (width) × 40 (length) × 16 (height) cm] in a room maintained at 23±2°C. Rats were kept in a 12-h light/dark cycle (lights on at 7:00 am) and allowed free access to food and water. All experiments were performed during the light phase. Each experimental group consisted of 6–10 animals. The experimental protocol was approved by the Ethics Committee for Use of Animal Subjects of the Faculdade de Ciências Farmacêuticas, UNESP (CEP-15/2010). Adolescence was defined as the age period between PND 28–42 (23).

### Drugs

The drugs used were testosterone (Pharma Nostra, Brazil; 10 mg/kg) dissolved in almond oil (vehicle) and fenproporex (Merck, Germany; 3 mg/kg) dissolved in 0.9% sterile saline.

### Behavior apparatus

The behavioral test was conducted in commercially available activity monitoring chambers (Columbus Instruments, USA), consisting of plexiglass cages. The chambers, measuring 45.1 (width) × 44.1 (length) × 8 (height) cm, contained 10 pairs of infrared photocells used to measure the horizontal locomotor activity. The consecutive interruption of two beams was recorded as one unit of locomotion.

### Effects of repeated testosterone administration on fenproporex-induced locomotor activity in adolescent and adult rats

The procedure started in PND 28 and the whole protocol took 13 days. From day 1 to 10, rats were weighed and given a subcutaneous (*sc*) injection of 10 mg/kg of testosterone or 1 mL/kg of vehicle, once a day. Immediately after the injections, animals were returned to their home cages. On days 11 and 12, animals did not receive any treatment. On test day 13 (PND 41), animals from the testosterone and the vehicle pretreated groups received intraperitoneal (*ip*) challenge injections of saline (1 mL/kg; VEH-SAL, n=8 and TEST-SAL, n=8) or fenproporex (3 mg/kg; VEH-FEN, n=10 and TEST-FEN, n=9). Animals were allowed 20 min of habituation and locomotor activity was recorded during 40 min.

Thirty days after the last testosterone injection (PND 68), the same animals again received the 10-day treatment with testosterone or vehicle, as described above. On day 13 (PND 81), the animals received another fenproporex (3 mg/kg, *ip*) or saline injection and their locomotor activity was recorded, as described above.

The dose of fenproporex (3 mg/kg) used in these experiments is known to induce locomotor activity in the absence of stereotypy behaviors (24,25). The dose of testosterone (10 mg/kg) was previously shown to change psychoactive-induced locomotor activity and is consistent with the abuse dosage used by humans (26,27).

The time period of 10 days for testosterone treatment was considered long enough to cause central plasticity in the reward system and promote locomotor sensitization (28).

### Statistical analyses

Data were analyzed by repeated measures three‐way ANOVA, using between-subjects factors of pretreatment (TEST or VEH), challenge (FEN or SAL) and time (5 to 40 min) as repeated factor. Newman-Keuls' test was employed for individual *post hoc* comparisons. Significant differences are reported for P<0.05.

## Results

### Adolescent rats

**Figure 1.**
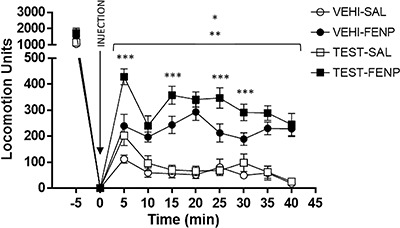
Locomotor activity following fenproporex (3 mg/kg, *ip*) challenge in adolescent rats (post-natal day, PND 41, n=35) pretreated with repeated testosterone injections (10 mg/kg, *sc*) from PND 28 to PND 37. Locomotor activity was measured during 40 min. Data are reported as means±SE. -5 min: habituation. *P*<*0.05: vehicle-fenproporex (VEHI-FENP) significantly different from vehicle-saline (VEHI-SAL). **P<0.05: testosterone-fenproporex (TEST-FENP) significantly different from testosterone-saline (TEST-SAL). ***P<0.05: TEST-FENP significantly different from VEHI-FENP (three‐way ANOVA followed by Newman-Keuls' test).

Figure 1 shows the locomotor activity following fenproporex (3 mg/kg, *ip*) challenge in adolescent rats (PND 41) pretreated with repeated testosterone injections (10 mg/kg, *sc*) from PND 28 to PND 37. ANOVA revealed significant differences in locomotor activity considering the factors pretreatment (F_(1,31)_=19.97; P<0.05), challenge (F_(1,31)_=247.90; P<0.05) and time (F_(7,217)_=6.19; P<0.05). In addition, a significant interaction was observed between the factors pretreatment and treatment (F_(3,31)_=6.09; P<0.05), between pretreatment and time (F_(7,217)_= 1.97; P<0.05) and between treatment and time (F_(7,217)_= 1.84; P<0.05).

Newman‐Keuls' *post hoc* test showed that fenproporex increased the locomotor activity in both vehicle and testosterone groups during the entire test (5 to 40 min) confirming the stimulant effect of fenproporex. Moreover, locomotor activity in response to a challenge injection of fenproporex was higher in the testosterone group compared to the vehicle groups at 5, 15, 25, and 30 min, indicating the presence of cross-sensitization between testosterone and fenproporex.

### Adult rats

**Figure 2.**
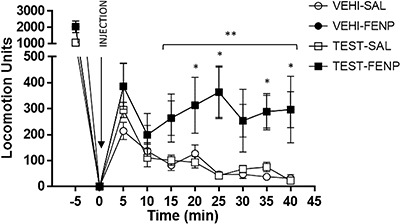
Locomotor activity following fenproporex (3 mg/kg, *ip*) challenge in adult rats (PND 81, n=24) pretreated with repeated testosterone injections (10 mg/kg, *sc*) from PND 68 to PND 78. Locomotor activity was measured during 40 min. Data are reported as means±SE. -5 min: habituation. *P<0.05: vehicle-fenproporex (VEHI-FENP) significantly different from vehicle-saline (VEHI-SAL). **P<0.05: testosterone-fenproporex (TEST-FENP) significantly different from testosterone-saline (TEST-SAL) (three‐way ANOVA followed by Newman-Keuls' test).

Figure 2 depicts the locomotor activity following fenproporex (3 mg/kg, *ip*) challenge in adult rats (PND 81) pretreated with repeated testosterone injections (10 mg/kg, *sc*) from PND 68 to PND 78. In adults rats, ANOVA did not reveal significant differences in locomotor activity induced by fenproporex considering the pretreatment factor (F_(1,20)_=0.57; P>0.05). However, a significant difference was found for the factors challenge (F_(1,20)_=22.87; P<0.05) and time (F_(7,140)_=4.26; P<0.05). An interaction between treatment and time factors (F_(7,140)_=4.26; P<0.05) was observed.

Newman‐Keuls' *post hoc* test showed that fenproporex increased the locomotor activity in animals pretreated with vehicle at 20, 25, 35 and 40 min and in animals pretreated with testosterone at 15, 20, 25, 30, 35 and 40 min, confirming the stimulant effect of fenproporex. However, repeated testosterone did not change fenproporex-induced locomotion in adult rats.

## Discussion

Based on recent reports and studies, we investigated the cross-sensitization between testosterone and fenproporex in adolescent and adult rats. Our results showed that repeated testosterone injections (10 mg/kg, *sc*) caused cross-sensitization to fenproporex (3 mg/kg, *ip*) in adolescent, but not in adult rats.

Behavioral sensitization is common to almost all the psychoactive drugs of abuse, and is related to stimulant addiction (29,30). Pre-treatment with a psychostimulant can increase or facilitate the behavioral response to the same drug (30,31). Behavioral sensitization was demonstrated to cocaine, amphetamine, nicotine, alcohol and heroin (30). Indeed, it has been demonstrated that all the psychoactive drugs act as positive reinforcement and promote behavioral sensitization (30,31). Similarly, cross-sensitization indicates that the exposure to a psychoactive drug could increase or facilitate the abuse vulnerability to another drug (32–34). Cross-sensitization was already demonstrated between nicotine and cocaine, amphetamine and nicotine and testosterone and cocaine (23,31).

Our results suggested that in adolescent rats, repeated testosterone treatment increased and/or facilitated the vulnerability to the stimulant effects of fenproporex. Moreover, the cross-sensitization between testosterone and fenproporex observed in the present study suggested that AAS induced neuroplasticities in neural pathways related to fenproporex psychomotor activation.

The behavioral sensitization reflects neuroadaptations in the dopaminergic limbic system that are involved in addiction (32). This system is formed by the ventral tegmental area (VTA) and its projections to the limbic system, which include the nucleus accumbens (NAc), amygdala and pre-frontal cortex (32,34). Studies have demonstrated neuroadaptations in the mesolimbic system after testosterone use. For instance, Birgner et al. (35) showed that nandrolone administration (3 ou 15 mg/kg) during 14 days in adult rats caused increases expression of D4 receptor RNAm in the NAc, decreases expression of D1 RNAm in the hippocampus, and increases expression of D1 RNAm in the amygdala. Moreover, other studies demonstrated that testosterone seems to have actions in the opioidergic neurotransmission. Indeed, Johansson et al. (36) showed that rats that received nandrolone (5 ou 15 mg/kg) during 14 days presented increased levels of β-endorphin in the VTA. Johansson et al. (37) also demonstrated increased levels of dynorphin and enkephalin in the hippocampus and striatum of rats.

In our study, we observed cross-sensitization in adolescent, but not in adult rats. Our results corroborated our previous study showing cross-sensitization between testosterone and cocaine in adolescent, but not in adult rats (22). However, in that study we used different groups for adolescent and adult rats. Here we re-treated the adolescent rats with testosterone in adulthood. Interestingly, in the present study, we found that the testosterone-induced cross-locomotor sensitization was not long lasting.

The neural basis related to amphetamines producing different behavioral outcomes between adult and adolescent rats is not fully understood. These age differences in stimulant sensitivity to testosterone and fenproporex have been attributed to ontogenetic changes in the dopaminergic system (38,39). For instance, it has been demonstrated that tyrosine hydroxylase expression varies during ontogeny. Moreover, changes in D2 receptor density is also observed during the maturation period (40).

Summarizing, repeated testosterone injections induced behavioral sensitization to fenproporex in adolescent rats, however this was not long lasting. Our results suggested that if repeated AAS treatment produces plasticities in the mesolimbic system of adolescent rats, these changes do not persist until adulthood. Future studies in animal models of adolescence are necessary to investigate this hypothesis and evaluate the molecular mechanisms related to testosterone and fenproporex cross-sensitization.
